# Survey of Teleneurology Use by Neurologists in a Low-Middle Income Country

**DOI:** 10.7759/cureus.53430

**Published:** 2024-02-01

**Authors:** Aliu O Yakubu, Chibuike Nwachukwu, Oreoluwa Morakinyo, Augustine C Amuta, Tobi N Olajide, Waliu Yakubu, Ayotomiwa Fagbemi, Luqman Ogunjimi

**Affiliations:** 1 Old Age Psychiatry, University Hospital Wishaw, Wishaw, GBR; 2 Breast Surgery, St George's University Hospitals NHS Foundation Trust, London, GBR; 3 Neurology, University of Texas Medical Branch, Galveston, USA; 4 Health and Wellness, Prince George's County Health Department, Upper Marlboro, USA; 5 Medicine and Surgery, College Research and Innovation Hub, Ibadan, NGA; 6 Medicine, University of Ibadan, Ibadan, NGA; 7 Nursing, Atiba University, Oyo, NGA; 8 Neurology, Federal Medical Centre, Abeokuta, Abeokuta, NGA; 9 Pharmacology and Therapeutics, Obafemi Awolowo College of Health Science, Olabisi Onabanjo University, Sagamu, NGA

**Keywords:** sub-saharan africa, teleneurology, telehealth, tele-consultation, low-middle income countries, neurology, nigeria

## Abstract

Background

Teleneurology has been in existence for decades, and the COVID-19 pandemic has escalated its widespread usage. Neurological conditions are a leading cause of death globally, with sub-Saharan Africa bearing the bulk of the burden. Nigeria has few trained neurologists with the few available concentrated in an urban region. The adoption of teleneurology will help close this treatment gap. Despite evidence of its advantage, the adoption and state of teleneurology in Nigeria are very low. This study aims to determine the state and perception of teleneurology in the care of neurological patients in Nigeria and identify challenges to its wide usage.

Methods

The primary research method was a descriptive cross-sectional survey among 48 neurologists in Nigeria across the six geo-political zones of the country. Descriptive statistics such as frequency and percentage were used to summarize and present the results.

Results

A total of 48 neurologists participated, of which 46 (95.8%) specialized in general neurology. Videoconferencing is the most preferred means of telemedicine (24, 50%), followed by phone calls (16, 33.3%) and short messages (6, 12.5%). Three-quarters of the respondents are concerned about legal actions from telemedicine use. The majority (34, 70.9%) are not familiar with telemedicine tools, and 40 (83.3%) indicate low telemedicine seminar attendance. More than 90% (46) of neurologists believe that it is a viable approach and can save time and money. Barriers to telemedicine included the lack of incentive to use the technology (38, 79.2%), poor Internet connectivity (36, 75%), and the lack of exposure to telemedicine (36, 75%).

Conclusions

It is important to overcome the existing barrier to teleneurology in order to fully harness its potential in addressing the shortage of health professionals in Nigeria as most neurologists are open to using it.

## Introduction

Although telemedicine has been in existence for many years, the emergence of the COVID-19 pandemic accelerated its adoption across the globe. Telemedicine is the use of technological devices to link patients to their providers to provide medical services such as diagnosis, treatment, and disease prevention [1]. It can occur in real time (synchronous) and store-and-forward (asynchronous) [1,2]. Real time is synchronous real-time communication between providers and patients via telephone, video-calling, or videoconferencing (usually achieved with the help of a patient assistant who conducts the prescribed examinations). Alternatively, store-and-forward is the practice of transmitting a patient's medical record across a long distance and does not require simultaneous attention from both the sender and the receiver. Smartphones, emails, and web servers can be used to transmit all forms of medical data such as medical imaging and laboratory test results.

The World Health Organization estimates that neurological conditions contribute to 6.3% of the global burden of disease, with Africa accounting for 2.9% of this fraction [3]. Nigeria has a population of 217 million [4], and there are only about 80 neurologists, resulting in a neurologist-to-population ratio of 1 to 2.7 million [5]. Furthermore, the few available neurologists are concentrated in the urban regions, resulting in sub-optimal treatment and unfavorable outcomes. Telemedicine in neurology, also known as tele-neurology, will play a key role in addressing the treatment gap and increasing access to quality healthcare.

Teleneurology has been successfully used in managing various neurological conditions, and its advantages include expanding practice reach, reducing travel time and costs for patients and doctors, facilitating educational opportunities and continuous medical education for physicians, and both one-on-one and group education of patients about their neurologic diseases [6,7]. On the African continent, teleneurology is developing quickly, creating opportunities for better medical treatment in underserved communities. For instance, in Ghana, telemedicine is being used for post-stroke blood pressure and medication review through a means of task shifting [8].

Telemedicine is not a new system in Nigeria, and as of 2019, there are 184 million mobile lines with 126 million connected to the Internet [9]. The Nigerian government initiated a hospital information and communication technology (ICT) strategic plan to implement telemedicine and ICT technology. Despite this, the lack of proper funding for telemedicine equipment by the government, the unwillingness of health workers to adopt this innovation, unstable Internet connections, and erratic electricity supply in many parts of Nigeria have hindered the widespread implementation of telemedicine [10].

Despite the rapid rise in the use of mobile health apps and awareness, especially after the coronavirus pandemic, there is limited literature on the application of telemedicine by neurologists and its use among patients with neurological diseases. Its use in neurological conditions is crucial due to the chronic and debilitating nature of the diseases, the need for consistent check-in and follow-up, and the ambulatory difficulty experienced by most neurology patients. This study aimed to determine the state and perception of teleneurology among neurologists in the care of neurological patients in Nigeria and identify challenges to its wide usage.

## Materials and methods

Methodology 

Study Design 

This was a descriptive cross-sectional study conducted among neurologists in Nigeria. There are currently about 80 practicing neurologists across the six geopolitical zones of the country [5]. Neurology consultants in different parts of the country who were available and willing to participate in the study, irrespective of age, gender, or years of experience, were recruited for the study. Exclusion criteria include neurology residents, non-neurology consultants, and retired neurologists. A convenience sampling technique (non-probability sampling) was utilized. Ethical approval was obtained from the Institute of Advanced Medical Research and Training, College of Medicine, University of Ibadan, Nigeria (approval number 22/0378).

Data Collection Techniques 

The participants were approached electronically through an electronic online survey. A standard questionnaire adopted from a previous study was used [11]. Written informed consent was obtained from the respondents. Emails were sent to the 80 neurologists to request their availability and willingness to participate in the study, out of which only 48 agreed. This yielded a response rate of 60%. The questionnaire consists of mainly five sections: (1) demographic characteristics, (2) access to computers and their literacy, (3) knowledge and perceptions, 4) barriers, and (5) willingness to adopt teleneurology.

Data Analysis

Data obtained were analyzed using Statistical Product and Service Solutions (SPSS, version 22; IBM SPSS Statistics for Windows, Armonk, NY). Descriptive statistics such as percentage and frequency were used for the result summary.

## Results

Table 1 shows the socio-demographic characteristics of participants. A total of 48 neurologists were surveyed, 34 (71.9%) were male, and 24 (50%) were between 30 and 39 years old. Most of the participants (46, 95.8%) specialized in general neurology, and 26 (54.2%) practiced in the southwest part of the country. Thirty-six (75%) of the participants were worried about medico-legal issues accompanying the use of telemedicine. The purpose of using the Internet varies, with 48 (100%) using it for literature search, 46 (95.8%) for upgrading knowledge and skills, 34 (70.8%) for getting information to give to patients, and 26 (54.2%) using it for consultation. Only 20 (41.6%) of the participants used tablets as telemedicine devices, while 46 (95.8%) and 38 (79.2%) used smartphones and personal computers, respectively.

**Table 1:** Socio-demographic characteristics of participants The data have been represented as N and %.

Variables	Responses	N	%
Age	30-39	24	50%
	40-49	16	33.3%
	Above 50	8	16.7%
Gender	Male	34	71.9%
	Female	14	28.1%
Location of practice	South-west	26	54.2%
	South-south	10	20.8%
	South-east	2	4.2%
	North-west	6	12.5%
	North-central	4	8.3%
Primary sub-specialty	General neurology	46	95.8%
	Epilepsy	2	4.2%
Concerned about legal actions	Yes	36	75%
	No	12	25%
Purpose of using social media	Getting information	34	70.8%
	Patient consultation	26	54.2%
	Literature search	48	100%
	Maintain your knowledge and skills	46	95.8%
Telemedicine device	Smart phone	46	95.8%
	Tablet	20	41.6%
	Personal computer	38	79.2%

**Figure 1:**
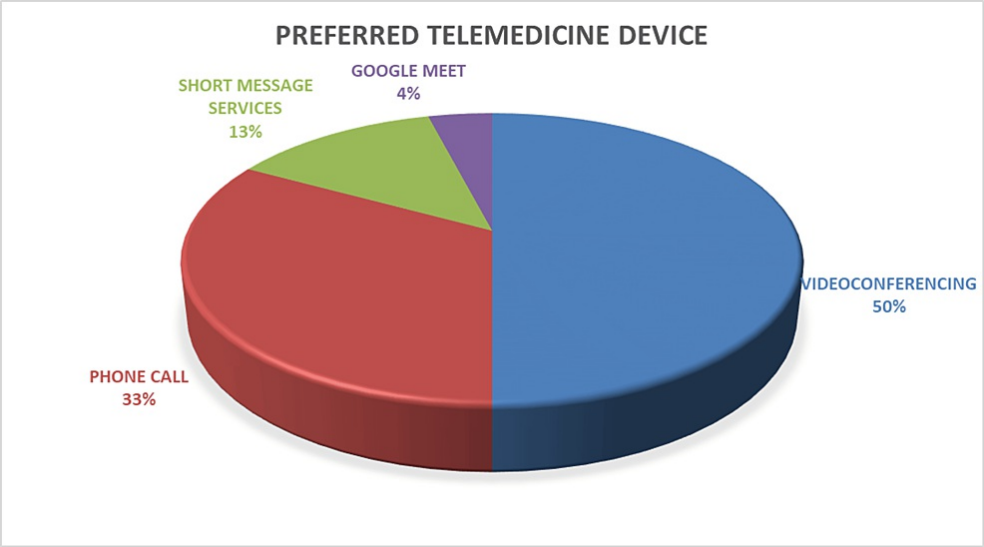
Participants' preferred telemedicine device The data have been represented as %.

Twenty-four (50%) preferred videoconferencing as a means of teleconsultation, followed by phone calls (16, 33.3%), short message services (6, 12.5%), and Google Meet (2, 4.2%; Figure 1).

Table 2 shows access to computers and literacy. The study showed that most (42, 87.5%) always used their laptop, and, interestingly, all the participants searched for information online.

**Table 2:** Access to computers and literacy The data have been represented as N and %.

Variables	Responses	N	%
How often do you use a PC/laptop?	Always/often	42	87.5%
	Sometimes/rarely	6	12.5%
How often do you search for information online?	Always	48	100%
In your role as a neurologist, how often do you interact with patients via e-mail or through social media?	Always	6	12.5%
	Sometimes/rarely	40	83.3%
	Never	2	4.3%
Have been asked by patients about online means of contact?	Always/often	16	33.3%
	Sometimes/rarely	26	54.2%
	Never	6	12.5%

Table 3 shows the knowledge and perception of teleneurology. One-third of the participants (33.3%) were not familiar with teleneurology. Only two (4.2%) were well-familiar with the medical applications of telemedicine, and the majority (34, 70.9%) were not familiar with telemedicine tools. Most (40, 83.3%) of the participants had never attended a telemedicine conference, and 36 (75%) of the participants were unfamiliar with telemedicine usage in other countries.

**Table 3:** Knowledge and perception of teleneurology The data have been represented as N and %.

Variables	Responses	N	%
To what extent are you familiar with teleneurology?	Low/never	16	33.3%
	Average	26	54.2%
	High	6	12.5%
To what extent are you familiar with the medical applications of telemedicine technology?	Low/never	26	54.2%
	Average	20	41.7%
	High	1	4.2%
How often do you attend conferences, speeches or meetings in your workplace regarding teleneurology?	Low/never	40	83.3%
	Average	4	8.3%
	High	4	8.3%
To what extent are you familiar with teleneurology tools?	Low/never	34	70.9%
	Average	12	25%
	High	2	4.2%
To what extent are you familiar with telemedicine guidelines?	Low	22	45.8%
	Average	12	25%
	Never	14	29.2%
To what extent are you familiar with the use of teleneurology in other countries?	Low/never	36	75%
	Average	12	25%
To what extent is continuous training training in the use of teleneurology necessary for neurologists?	Never	26	29.5%
	Average	12	41.7%
	High	10	29.2%

Table 4 shows the participants' knowledge of barriers to telemedicine implementation. Barriers to telemedicine included a lack of incentive to use the technology (38, 79.1%), poor Internet connectivity (36, 75%), and a lack of exposure to telemedicine (36, 75%). Only one-quarter (25%) believe that it is difficult to use, and 10 (20.9%) think patients will not like telemedicine.

**Table 4:** Participants' knowledge of barriers to the implementation of teleneurology The data have been represented as N and %.

Variables	Agree, n (%)	Disagree (%)
Patients do not like telemedicine	10 (20.9%)	38 (79.1%)
Neurologists do not like telemedicine	14 (30%)	34 (70%)
Physicians lack incentives to use telemedicine	38 (79.1%)	10 (20.9%)
Telemedicine is not reliable	10 (20.9%)	38 (79.1%)
Telemedicine is difficult to use	12 (25%)	36 (75%)
Internet connectivity is a major challenge	36 (75%)	12 (25%)
Telemedicine is difficult to understand	8 (16.6%)	40 (83.4%)
There is a lack of exposure to telemedicine	36 (75%)	12 (25%)
The technology required for telemedicine is expensive	18 (37.5%)	30 (62.5%)
Telemedicine is not as effective as in-person consultations	36 (75%)	12 (25%)
Telemedicine is limited to certain geographic areas	24 (50%)	24 (50%)
There are concerns about patient privacy/confidentiality	30 (62.5%)	18 (37.5%)
There is a high cost of equipment to effectively use telemedicine	36 (54.2%)	22 (45.8%)

**Figure 2:**
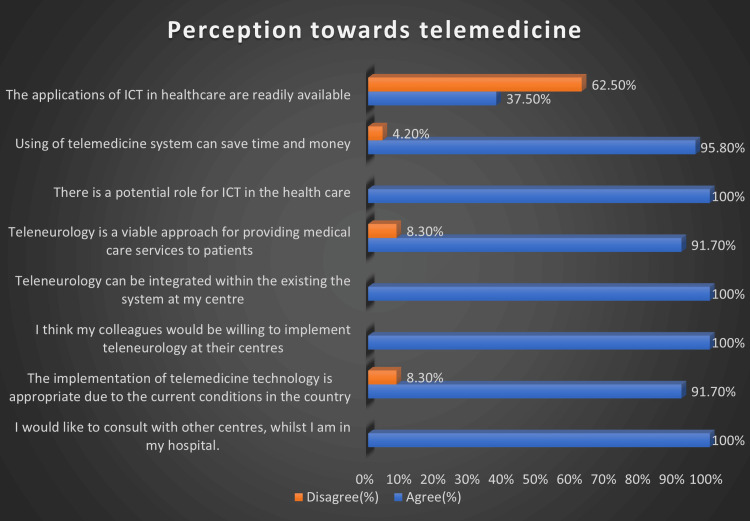
Participants' perception towards telemedicine use The data have been represented as %.

Figure 2 depicts the perception towards telemedicine. Forty-six (95.8%) of the neurologists believe that it is a viable approach and can save time and money, whereas 30 (62.5%) believe that the application of information and communication technology is not readily available. All our respondents agreed that ICT has a potential role in healthcare. All our participants agreed that telemedicine could be integrated into the healthcare system and were open to consulting with other centers via the use of telemedicine devices.

## Discussion

Gender distribution within the field appeared to be skewed, with a notable 34 (71.9%) of respondents being male, and 14 (28.1%) being female respondents. A study in the US showed a similar trend, revealing that gender disparity and male preponderance among healthcare providers have been on the rise in our healthcare system, particularly in some specialties such as vascular neurology [12]. Regarding geographical distribution, the southwest region had 26 (54.2%) respondents, which is similar to a previous study that showed a high concentration of doctors in the southwest region of Nigeria [13]. Concerning telemedicine device preferences, the study revealed that smartphones were the most favored choice, closely followed by personal computers and then tablets. Another study showed that laptops are the most used device (115, 82.1%), followed by smartphones (95, 67.9%) and tablets (45, 32.1%) [14]. Mobile devices are slowly replacing laptops due to their combining the features and functions of mobile phones with advanced computing capabilities, enabling users to access a wide range of software applications [11].

In the context of preferred telemedicine devices, videoconferencing emerged as the top choice for telemedicine consultations, selected by 24 (50%) neurologists. This finding is similar to a study among Philippine neurologists in which real-time communication is preferred over stored-and-forward, as videoconferencing is preferred by 122 (87.1%) [14]. Many neurologists appreciated the multi-functionality of videoconferencing platforms, often equipped with screen-sharing capabilities, and collaborative tools, allowing them to perform physical examinations for better decision-making. These features enhanced the consultation process and facilitated collaborative discussions among medical teams. For some, text-based communication via short message services (SMS) may have been seen as a convenient and less intrusive option, suitable for quick exchanges and sharing updates. A smaller percentage (2, 4.2%), opted for Google Meet, possibly driven by the desire for professional collaboration. This platform not only served patient consultations but also supported inter-colleague discussions, case reviews, and educational purposes. Neurologists may have considered patient preferences, regulatory compliance, and local infrastructure when choosing telemedicine platforms. Patients' comfort and technological proficiency may have influenced platform selection, and concerns about regulatory compliance and data security may have played a role. Moreover, the availability and quality of Internet and communication infrastructure in the region where neurologists practice could have significantly impacted their choices. In areas with limited Internet access, simpler options such as phone calls may have been favored. Ultimately, it is likely that neurologists made these choices based on the individual needs of their patients and the specific requirements of their practices. It is noteworthy that 36 (75%) respondents expressed concerns about legal issues associated with telemedicine. Previous studies have shown that physicians are concerned about the medico legal and ethical issues with telemedicine usage [11,15].

Furthermore, all our respondents actively searched for information online, showcasing their commitment to staying informed and up-to-date through digital resources. This is in contrast with a study in Saudi Arabia where only four (3.1%) searched the Internet for information [11]. This difference might be due to the regional perception of the usage of Internet sources for clinical knowledge. In the realm of patient interaction, a significant number (46, 95.8%) of neurologists engaged with patients through email and social media. Social media is an effective means of communication with patients [16]. This underscored the evolving landscape of patient-physician interaction, reflecting the increasing role of digital channels in healthcare communication. More than four-fifths (42) of the respondents reported being asked by patients about online means of contacting them. This observation demonstrated a growing patients’ interest in virtual communication with healthcare providers.

More than half of our respondents (n=26; 54%) were unfamiliar with the medical application of telemedicine as only one person (4.2%) was conversant with the use of teleneurology. A study among neurologists in the UK demonstrated good awareness and use of teleneurology, citing some challenges of its use for neurology patients [17]. This difference in awareness could be due to the difference in study setting as our study setting was in Nigeria, which is a developing country with limited resources to fund telemedicine. A study in Nigeria among 158 healthcare providers (HCP), through an online survey, showed that 142 (90%) of HCPs reported that they used some form of telemedicine, and 98 (62%) believed that it would improve healthcare consultation experience and satisfaction citing affordability, time-saving, and safe delivery of healthcare during pandemics as some of its advantages [18]. These findings are at variance with the findings from our study where 16 (33%) of the respondents had little or no knowledge of teleneurology. This could be because the index study had respondents including doctors in training, interns, and consultants across all fields, but our study focused on consultant neurologists who may not have been exposed to telemedicine during their training.

Twenty-six (54.2%) of our respondents were unfamiliar with the medical applications of telemedicine. However, in a study done in Enugu, Nigeria, most of the respondents (146, 98%) had heard about telemedicine, but only 100 (67.3%) had consulted using telemedicine [15]. This study was a multispecialty study among doctors, and this could account for the variance in results. The first Arab-Africa teleneurology conference was held in Egypt in 2016, to advance the knowledge and use of teleneurology [19]. Only eight (16.6%) of our respondents had attended such a conference. This could contribute to the limited knowledge and use of tele-neurology among neurologists in Nigeria. In a study done among doctors, nurses, pharmacists, and physiotherapists in southwest Nigeria, only 13(13%) of the respondents had attended a workshop on telemedicine, and about 87(80%) of them believed that training was necessary for the advancement of telemedicine practice [20]. About 10 (29.2%) of our respondents believed that training in teleneurology was necessary for neurologists. Our study was conducted among consultants who probably have been trained without telemedicine and have been practicing without telemedicine and so have been used to clinical practice without the use of telemedicine. This could account for the relatively low percentage of respondents from our study who were in favor of training for teleneurology. Impressively, all respondents expressed their interest in consulting with other centers while remaining in their own hospitals, indicating a strong desire for collaborative and remote healthcare, and believed that their colleagues would be willing to implement teleneurology at their centers. Moreover, 44 (91.7%) neurologists found the implementation of telemedicine technology appropriate, recognizing its potential in the current healthcare landscape. This willingness is similar to a previous one in Nigeria where it shows acceptability for the use of telemedicine for consultation by physicians [18]. This underscored the adaptability of telemedicine technology and its potential to enhance the healthcare infrastructure.

Forty-four (91.7%) of our respondents agree that teleneurology is a viable approach to providing healthcare. Similarly, a study done in the United States revealed the potential of telehealth in providing health services, and this led to more funding and policy-making to improve the use of telemedicine in providing neurological services to patients [21]. Studies have shown the gains of the use of tele-neurology in the management of Parkinson's and stroke cases [22-24]. All participants from our study agreed that ICT has potential in healthcare, and this was demonstrated during the COVID-19 pandemic when technology was deployed in patient care [25,26]. Forty-six (95.8%) of our respondents believed that telemedicine use can save time and money. This was also reported in a study done in the United States on the use of telemedicine among cancer patients, and it was found to save time as well as money to the tune of 140-178 dollars per visit [27].

According to a study done in Saudi Arabia among doctors, nurses, and other health workers, the barriers to the use of telemedicine include poor Internet connection (372, 36%), lack of knowledge about telehealth (372, 36%), lack of trained staff (320, 31%), and lack of expert support (300, 29%) [28]. Our study also revealed challenges such as difficulty in using telemedicine (12, 25%), Internet connectivity problems (36, 75%), lack of incentive (38, 79.2%), and willingness to use telemedicine (14, 30%). Despite the difference in study location, respondents, and sample size, similar challenges were reported, although with slight differences in proportions as their study had over 1,000 participants in various fields, while our participants were less than 100 and consisted mainly of neurologists. A systematic literature review of data from different parts of the world done in 2016 revealed issues with cost, confidentiality, and effectiveness of telemedicine as challenges [29]. Similarly, our study highlighted the challenges of cost (n=26; 54.2%), effectiveness (n=36; 75%), and confidentiality (n=30; 62.5%).

Limitations 

This cross-sectional study relied on self-reported data that were subjected to recall bias, which could have overestimated or underestimated the actual results. The convenience sampling technique used in this study may introduce some degree of bias. The sample size was small due to the limited number of neurologists in Nigeria, which also limited the statistical analysis of the data. A robust study among neurologists in Africa will give a broader view of neurologists across the continents.

## Conclusions

This research report provided valuable insights into the readiness of neurologists to embrace telemedicine and digital practices. It underscored the importance of addressing legal concerns and the need for regulatory support. The media can raise awareness about the benefits and accessibility of teleneurology, while the government can implement policies and invest in infrastructure to support its widespread adoption, ultimately fostering a more inclusive and efficient healthcare system.

Moreover, it highlighted the pivotal role of digital tools in staying informed and providing patient care in the evolving landscape of neurology. This research contributed to the ongoing discourse on telemedicine adoption within the field of neurology and provided a foundation for further studies and interventions in this domain.
